# Single copy-sensitive electrochemical assay for circulating methylated DNA in clinical samples with ultrahigh specificity based on a sequential discrimination–amplification strategy†
†Electronic supplementary information (ESI) available. See DOI: 10.1039/c7sc01035d



**DOI:** 10.1039/c7sc01035d

**Published:** 2017-05-18

**Authors:** Xuyao Wang, Feng Chen, Dexin Zhang, Yue Zhao, Jing Wei, Lihua Wang, Shiping Song, Chunhai Fan, Yongxi Zhao

**Affiliations:** a Key Laboratory of Biomedical Information Engineering of Education Ministry , School of Life Science and Technology , Xi’an Jiaotong University , Xianning West Road , Xi’an , Shaanxi 710049 , P. R. China . Email: yxzhao@xjtu.edu.cn; b Department of Respiratory Medicine , The Second Affiliated Hospital of Medical College , Xi’an Jiaotong University , Xiwu Road , Xi’an , Shaanxi 710049 , P. R. China; c Division of Physical Biology , Bioimaging Center , Shanghai Synchrotron Radiation Facility , CAS Key Laboratory of Interfacial Physics and Technology , Shanghai Institute of Applied Physics , Chinese Academy of Sciences , Shanghai 201800 , P. R. China

## Abstract

Tumor-related circulating methylated DNA represents only a small fraction of the total DNA in clinical samples (*e.g.* plasma), challenging the accurate analysis of specific DNA methylation patterns. Yet conventional assays based on the real-time quantitative methylation-specific PCR (qMSP) are generally limited in detection sensitivity and specificity due to its non-specific amplification interference including primer dimers and off-target amplification. Here we propose a single copy-sensitive electrochemical assay for circulating methylated DNA with ultrahigh specificity on the basis of a sequential discrimination–amplification strategy. Methylated DNA rather than unmethylated DNA in a bisulfite-modified sample is identified and amplified by the asymmetric MSP to generate abundant biotin-labeled single-stranded amplicons with reduced primer–dimer artifacts. Self-assembled tetrahedral DNA probes, which are readily decorated on an electrode surface as nanostructured probes with ordered orientation and well controlled spacing, enable the highly efficient hybridization of the specific single-stranded amplicons due to greatly increased target accessibility and significantly decreased noise. The interfacial hybridization event is quantitatively translated into electrochemical signals utilizing an enzymatic amplification. The proposed assay integrates dual sequence discrimination processes and cascade signal amplification processes, achieving the identification of as few as one methylated DNA molecule in the presence of a 1000-fold excess of unmethylated alleles. Furthermore, the excellent assay performance enables tumor related methylation detection in lung cancer patients with 200 microlitre plasma samples. The results are in good consistency with those of clinical diagnosis, whereas the conventional qMSP failed to detect the corresponding methylation pattern of these clinically confirmed positive patients in such trace amounts of samples.

## Abstract 2

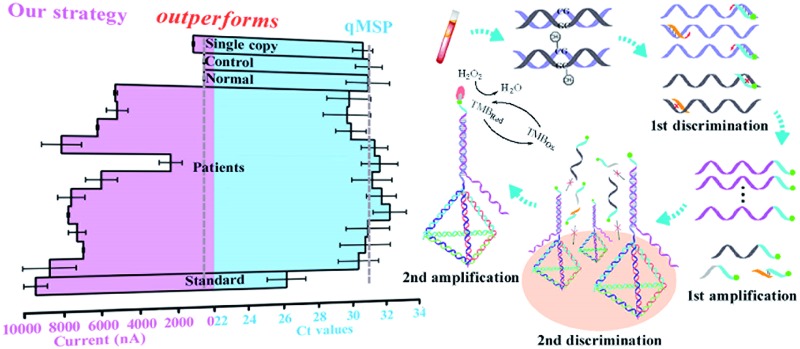
A sequential discrimination–amplification (SEDA) electrochemical strategy was constructed for the identification of single-copy circulating methylated DNA.

## Introduction

DNA methylation is widespread in mammals and observed mainly as the addition of a methyl group to cytosines at CpG dinucleotides. The methylation of the promoter region CpG islands in tumor suppressor genes is frequently associated with the progression of cancers such as lung cancer.^1,2^ Accumulated evidence has indicated that methylated DNA could be released into circulation during different stages of the tumor, and this cell-free DNA can be regarded as a prognostic indicator for early cancer detection and behavior monitoring.^2,3^ However, tumor related methylated DNA represents only a small fraction of the total DNA in complex clinical samples (*e.g.* plasma), posing persistent technical challenges in the accurate analysis of specific DNA methylation patterns.

Numerous available tools to assess DNA methylation have been successively developed over the past two decades.^4–13^ Generally, three different strategies including sodium bisulfite conversion,^9^ restriction enzyme digestion^10–12^ and affinity enrichment^13^ are employed to identify methylated cytosines (^m^C) from unmethylated cytosines (C). Notably, the bisulfite-assisted methylation-specific PCR (MSP) has been one of the most common methylation analysis tools. This technique relies on the sodium bisulfite treatment of DNA, which converts C to uracils while leaving ^m^C unaffected, thus turning the epigenetic difference into the sequence difference. The modified DNAs are then amplified by the PCR with methylation-specific primers, the products of which are identified *via* gel electrophoresis. However, this MSP approach offers only qualitative analysis and low assay sensitivity. The real-time quantitative MSP (qMSP)^14,15^ takes advantage of the fluorescent dye reporter or TaqMan probe to achieve quantitative analysis, and improves the sensitivity. Nevertheless, it still often suffers from non-specific amplification including primer dimer formation and off-target amplification of unmethylated alleles.^16^ Besides the real-time qMSP, several novel end-point MSP strategies have been developed to solve these problems.^17–21^ Notably, the method (methylation-specific quantum dot fluorescence response energy transfer, MS-qFRET) reduced the number of PCR cycles required to inhibit the formation of primer dimers and pushed the detection limit of methylated DNA down to 15 pg.^19^ However, off-target byproducts from unmethylated DNA amplification still exist and can be detected by FRET as interfering signals.^19,22^ Nie and coworkers employed a supercharged green fluorescent protein as a versatile probe for the detection of MSP products, achieving the highly sensitive detection of methylated DNA extracted from human colon carcinoma tissue samples.^21^ Furthermore, this method used toehold strand displacement hybridization to improve the specificity by single-base mismatch between methylation and unmethylation sequences. In addition, Li’s group has developed an alternative methylation assay based on the ligase chain reaction. This assay evaded the problems of primer dimers and off-target amplification in the MSP, achieving the determination of as low as 10 aM and 0.1% methylated DNA from a large excess of unmethylated DNA with multiplexed methylation sites.^23^ Yet, mismatch ligation^24^ and blunt-end ligation also exist in the ligase chain reaction, causing non-specific signals. Thus, developing a highly specific and sensitive methylated DNA detection strategy without non-specific amplifications still remains a challenge.

Considering that the high concentration of the primers is always accompanied by the generation of primer dimers,^25^ we speculate that decreasing the concentration of the primers can reduce and even eliminate the dimers, for example, in the asymmetric PCR. However, it conversely suffers from low amplification efficiency. Thus the integration of a downstream amplification strategy is required to further improve the sensitivity.^26,27^ Additionally, as off-target amplification still remains in the asymmetric PCR, the combination of specific downstream discrimination can further improve the specificity.^28–31^ Recently, we developed a tetrahedral DNA nanostructure-based electrochemical detection strategy.^32,33^ These tetrahedrons provide greatly increased target accessibility and significantly minimize non-specific adsorption. By simultaneously utilizing enzymatic amplification, we realized the detection of nucleic acids in the aM-level. Collectively, by integrating the above solutions, we expect to overcome the problems of primer dimer formation and off-target amplification, realizing methylation detection with high sensitivity and high specificity.

Herein, we developed a sequential discrimination–amplification (SEDA) strategy for circulating methylated DNA detection with single-copy sensitivity and ultrahigh specificity. In this method, methylated DNA rather than unmethylated alleles in a bisulfite-modified genomic sample was identified and amplified by the asymmetric MSP (AMSP) using a biotin-labeled methylation-specific primer, producing lots of biotin-labeled single-stranded amplicons with reduced primer–dimer artifacts. DNA nanostructured probes *via* decorating self-assembled tetrahedral DNA on a gold electrode surface were employed to capture these amplicons. These probes greatly increased target accessibility and minimized the non-specific adsorption of amplification byproducts due to the rigid scaffold, ordered orientation and well controlled spacing, thus enabling the high efficiency and specific hybridization and significantly decreasing noise. Horseradish peroxidase-conjugated avidin (avidin–HRP) was then captured *via* biotin–avidin binding to generate an electrochemical signal by the enzyme catalytic process. Dual sequence discrimination processes, including methylation-specific annealing and specific interface hybridization, as well as cascade signal amplification processes represented by the asymmetric MSP and HRP catalytic reaction, were well integrated in the proposed assay, realizing the detection of single-copy methylated alleles. Moreover, the DNA methylation at the p16INK4a gene promoter in trace amounts of plasma samples (200 microlitres) from eleven lung cancer patients was accurately determined by our proposed strategy, the results of which are in good consistency with those of clinical diagnosis.

## Results and discussion

**Fig. 1:**
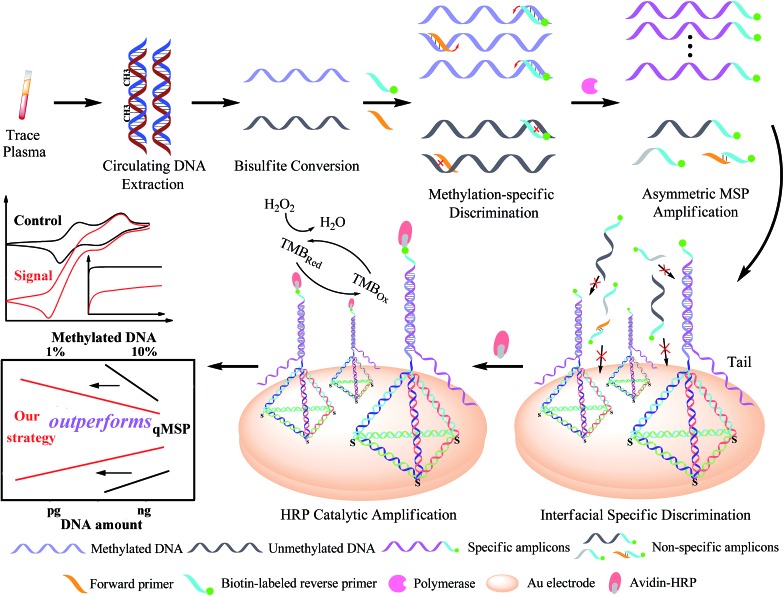
Schematic representation of the SEDA strategy. The biosensing is integrated by dual sequence discrimination processes including methylation-specific annealing and specific interface hybridization, as well as cascade signal amplification processes represented by the asymmetric MSP and HRP catalytic reaction. TMB_Red_ and TMB_Ox_ represent the reduction and oxidation states of 3,3′,5,5′-tetramethylbenzidine respectively.

The design principle of the proposed SEDA strategy is illustrated in Fig. 1. Circulating DNA was firstly extracted and converted with sodium bisulfite treatment. Methylation-specific primers were then applied to discriminate the methylated sequence. Through AMSP amplification, abundant specific amplicons of single-stranded DNA (ssDNA) were produced, accompanied by possible non-specific amplicons including off-target amplicons and primer dimers. Subsequently, interfacial DNA nanostructured probes were employed to discriminate specific amplicons from the possible non-specific ones. These probes are complementary to the region near the primer binding site in the specific amplicons. The hybridization sequence length is optimized from our previous work to guarantee the effectiveness as well as the specificity. Non-specific adsorption to the electrode surface is significantly minimized. Finally, avidin–HRP was introduced into the system, catalyzing the second signal amplification. The synergetic combination of dual sequence discrimination and cascade signal amplification decreased the interference from non-specific amplification and adsorption, and increased the detection sensitivity and specificity.

According to the chronocoulometric quantitation method reported,^34^ using a cationic redox marker, RuHex, we can prove the immobilization of the DNA tetrahedron on the electrode and further calculate the surface density (Fig. S1†) to be 4.2 × 10^12^ molecules per cm^2^, which corresponds to around 0.2 pmol on the 2 mm-diameter gold electrode. Using 200 nM of reverse primer as the excess primer, we can obtain at most 3.6 pmol of the ssDNA target amplicon (excluding 0.4 pmol of the double stranded amplicon). A definite amount of tetrahedral probe can theoretically hybridize with an equal amount of amplicon. Therefore, 1 μl of the reaction solution may be approximately saturated. To validate this, we further tested 3 μl of the solution and the result in Fig. S2† shows that the current only increases by 10% of that of 1 μl. Thus, the AMSP product is substantially excessive and when optimizing the experimental conditions, we diluted the AMSP solution 10-fold.

**Fig. 2:**
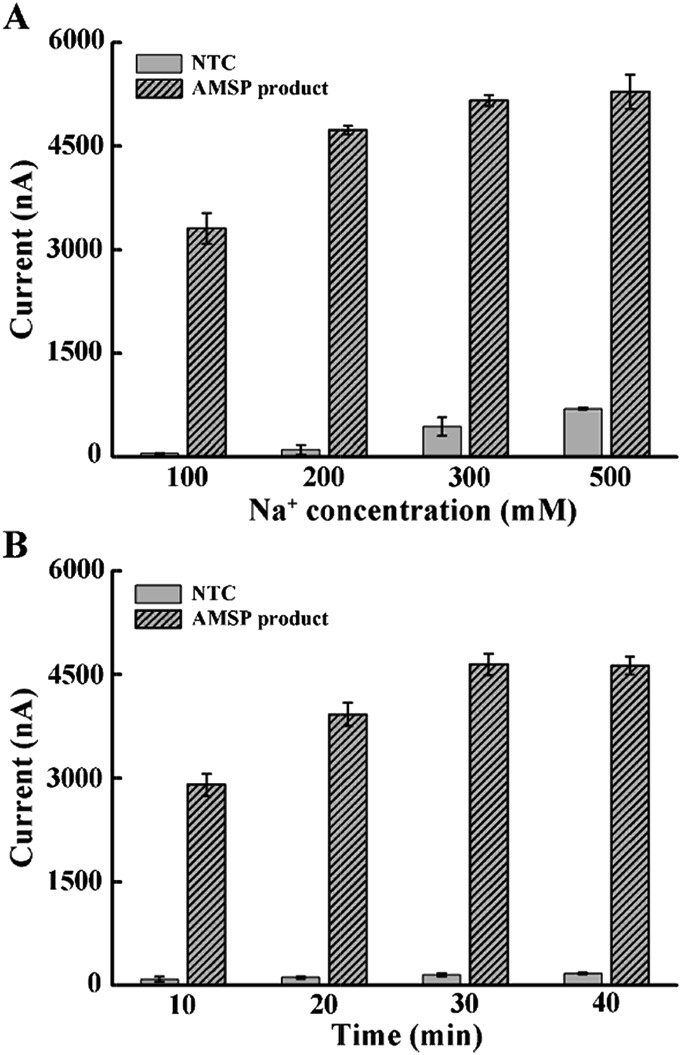
Optimal conditions for the hybridization between the tetrahedral probe with the ssDNA target. (A) The effect of Na^+^ concentration on DNA hybridization. (B) Incubation time of the DNA hybridization reaction. The error bars represent the standard deviation of the three measurements.

Considering that the probe is relatively long and that high ionic strength can help hybridization, we explored the effect of increasing the Na^+^ concentration in the hybridization buffer.^35^ As shown in Fig. 2A, the addition of Na^+^ in the hybridization system induced rapid background growth, whereas relatively slow signal growth was observed. Therefore, in order to avoid the impact of an excessive background, we chose 200 mM of Na^+^ for the following experiment. In addition, we tested the hybridization time and concluded that 30 min is enough for the reaction (Fig. 2B).

**Fig. 3:**
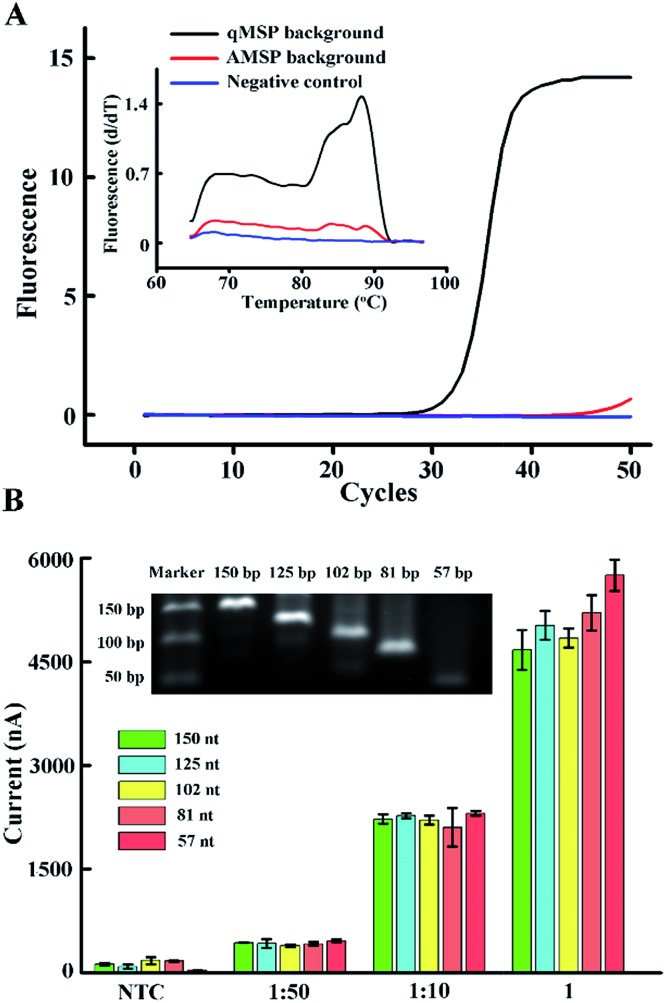
(A) Real-time fluorescence PCR curves and melting profiles of the qMSP background and AMSP background. The ratios of the two primers are 10 : 10, 1 : 10 and 0 : 10. (B) Current values affected by strand hybridization with different concentrations of the target ssDNA and different lengths of tails. The different concentrations are obtained by diluting the bulk AMSP solution 50 times (1 : 50) and 10 times (1 : 10). The bars labelled with NTC represent the non-template control (not diluted). The different lengths of the tails are shown with different colors. The inset shows the gel readout of the different lengths. A prediction of the secondary structure is shown in Fig. S4.† The error bars represent the standard deviation of the three measurements.

According to the design of the proposed hybridization strategy, we used a 34 nt probe to capture the 150 nt amplicon, yielding an overly long 3′-tail (the tail in Fig. 1). Due to the possible steric effect and electrostatic repulsion between the long tail and the skeleton of the tetrahedral DNA, we systematically studied the effect of different tail lengths on hybridization. We designed a series of forward primers and synthesized a template strand. All five single-stranded amplicons (inset of Fig. 3B, present in dsDNA) contain a region complementary to the tetrahedral probe, yielding a respective 93, 68, 45, 24 and 0 nt (non) tail. The effect of the primer sets in the qMSP is shown in Fig. S3.† We can see that when template DNA is present (positive), the Ct values are relatively lower, but in the absence of template DNA (negative), the curves still rise as the primer dimer forms, which is inevitable especially in the conventional MSP. However, when performing the AMSP, the background is much lower (Fig. 3A). As for the signal in the electrochemical measurements when diluting the PCR solution (stored as a stock solution) 10-fold, the difference of the current values caused by the different length of the tails responding to hybridization is negligible. Considering that the steric effect and electrostatic repulsion may be affected by the target concentration, we additionally tested the stock solution and the solution diluted 50-fold. The further diluted solution showed a similar phenomenon but the stock solution seemed slightly different. This may be thanks to the special construction of the tetrahedron that contains four sloping rigid triangles of DNA helices as the side face, with every two terminals of the oligonucleotides merging at each vertex. Under this circumstance, while this homogeneous self-assembled monolayer is neatly ordered in a relatively close arrangement at the bottom, the upper space is oppositely spacious. Thus, when target ssDNA is limited (dilution ratios of 1 : 10 and 1 : 50 in Fig. 3B), the dissociative tails in the relatively large buffer zone will not induce the steric effect and electrostatic repulsion between targets and targets with the tetrahedrons, which in turn does not interrupt or interfere with hybridization. However, when the targets are saturated (dilution ratio of 1 in Fig. 3B), longer tails will be affected more, but not enormously, and the factors of AMSP should also be taken into account as the presented electrochemical signal is a combination of the sequential discrimination–amplification processes. Furthermore, the non-tail target generated a very large current signal in high concentration, but no significant increase in limited concentration. Consequently, the original set of the primer which generates a long 150 nt target can be used in this strategy with no more consideration, as long as the target for hybridization is not saturated (which can be judged from the current value).

**Fig. 4:**
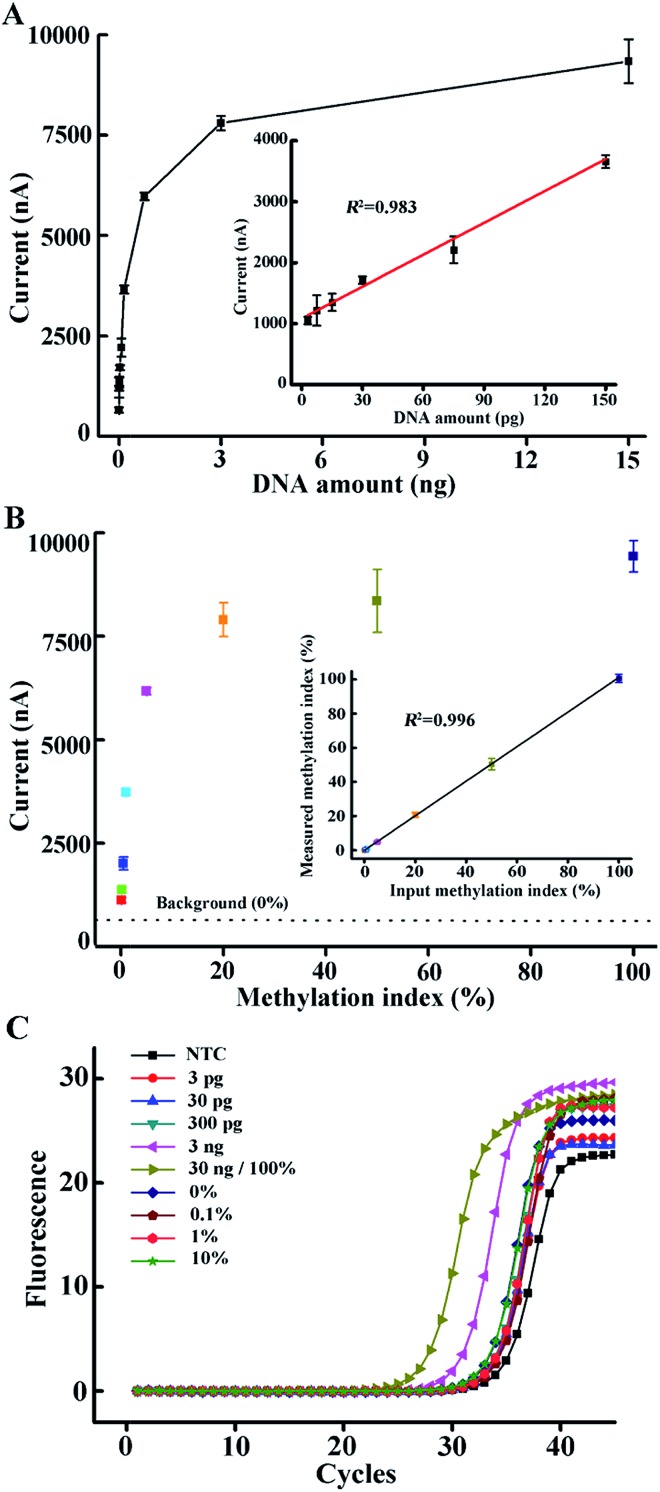
(A) Electrochemical current as a function of the amount of methylated DNA input. The corresponding current responses are shown in Fig. S5.† The inset shows the linear correlation between the current value and the amount of methylated DNA input in the range of 3–150 pg. (B) Electrochemical current as a function of the input methylation index in the mixtures of methylated and unmethylated DNA. The corresponding current responses are shown in Fig. S6.† The inset shows the linear correlation between the measured and the actual input methylation index in this artificial mixture of methylated and unmethylated DNA. The error bars represent the standard deviation of the three measurements. (C) qMSP curves of different DNA input amounts and different methylation indices.

In order to meet the critical demand for the diagnosis of methylation-related diseases, a good S/B ratio and great sensitivity for the analysis of an amount of DNA input as low as possible should be achieved. Here, we investigated the variation of the chronoamperometry current with the concentration of methylated DNA input. We used 1 μl of the AMSP product to produce a strong enough current so as to maintain the potential discrimination when an extremely low amount of DNA was added. As can be seen from Fig. 4A, methylated DNA down to a single copy can be discriminated from the NTC (non-template control), which means that once a correspondingly lower amount of target ssDNA is amplified, it can be captured by the stable DNA nanostructured probe system and presented as the further amplified current signal by the TMB–HRP system. Meanwhile, a linear correction (inset of Fig. 4A, *R* ^2^ = 0.983) can be observed when the DNA amount is relatively low. When the DNA amount was increased, an exponential curve was observed, which indicates that more and more tetrahedral probes were occupied and the second amplification of the signal continued. A plateau effect is nearly reached when a common ng-level of DNA was used as the template in the AMSP, which corresponds with the almost saturated probes. Compared to the conventional qMSP which usually has ng-level sensitivity (Fig. 4C), we expanded this method by analyzing a very large range of methylated DNA inputs (by arbitrarily diluting the AMSP product when a relatively large amount of DNA was added), and it has the potential to detect very low amounts of methylated DNA (down to a single copy that can be discriminated from the NTC).

Furthermore, the methylation index across a target region can vary among cancer types and stages of the disease.^36^ Clinical samples usually also contain a mixture of tumor and normal cells, leading to a mixture of methylated (M) and unmethylated (U) DNA, where the presence of unmethylated sequences can potentially influence the sensitivity and the specificity of the diagnostic assay.^37^ Particularly, for early cancer detection, it is essential to discriminate the low amount of methylated DNA from a high background of unmethylated DNA. Therefore, we investigated the ability of our approach to detect the DNA methylation index by mixing artificially methylated DNA with unmethylated DNA (a converted sequence from healthy volunteers) in a total amount of 15 ng (assuming 100% recovery in the conversion process) at different proportions representing 0, 0.1, 0.2, 0.5, 1, 5, 20, 50 and 100%. As shown in Fig. 4B, the chronoamperometry current increased with the methylation index. In the case of 0%, there was no methylated sequence present in the solution, and consequently, no methylation-specific primer-based extension occurred, and no sequence specific hybridization formed. The low background is caused by the non-specific binding between the biotin-labeled single strands (no extended reverse primers or primers extended in a non-specific way, nonetheless lacking the ability to hybridize with the tetrahedral probes, *i.e.* primer dimers) and the exposed gold electrode surface (Fig. S9†). Similar to the situation when only methylated DNA is present, the whole process successively consists of an exponential phase (the initial phase in the AMSP when both the forward and reverse primers exist), a linear phase (the latter phase in the AMSP when only the excess reverse primer exists) and a non-linear phase of target–probe hybridization and HRP-catalyzed TMB redox activity, resulting in an amperometric current.^38^ Thus, according to the curve obtained from the situation when only methylated DNA was present, we calculated the methylation index derived by the measured current value (*Y*-axis). The inset of Fig. 4B shows the plot of the measured methylation index against the actual input methylation index. A good correlation coefficient (*R* ^2^ = 0.996) was observed, proving that the presence of the unmethylated sequence does not interfere with the target methylated sequence detection in our approach. Meanwhile, the overly excessive unmethylated DNA interferes with the result obtained from the qMSP (*e.g.* the magenta curve compared to the green curve in Fig. 4C). Conversely, the high specificity reaching a 0.1% methylation index in our approach thus guarantees the ability to analyze a real sample even from early cancer patients.

**Fig. 5:**
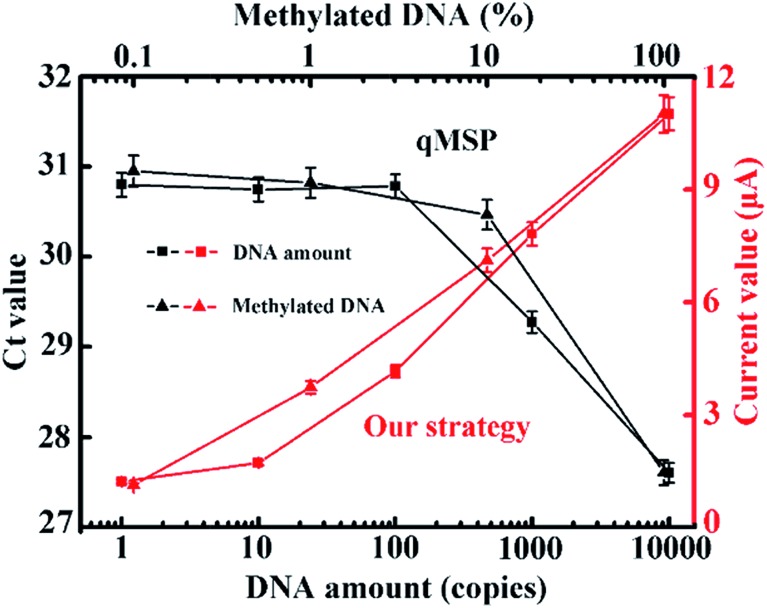
Comparison between the qMSP and our strategy with different amounts of methylated DNA input and different input methylation indices. The corresponding fluorescence curves and melting profiles are shown in Fig. S7 and S8.† Our strategy has obvious advantages in terms of sensitivity and specificity.

At this point, we have achieved the identification of as few as one methylated DNA molecule in the presence of a 1000-fold excess of unmethylated alleles. We collectively compared our method with the conventional qMSP. As can be seen in Fig. 5, only a ng-level (300 copies) of the DNA and 5% (20-fold) of the methylated DNA can be detected in the qMSP. Additionally, a reduced number of PCR cycles is enabled when coupling with this electrochemical method (Fig. S10†). These advantages are achieved by the synergetic combination of dual sequence discrimination and cascade signal amplification, which significantly decreased the interference from non-specific amplification and adsorption, and increased the detection sensitivity and specificity.

**Fig. 6:**
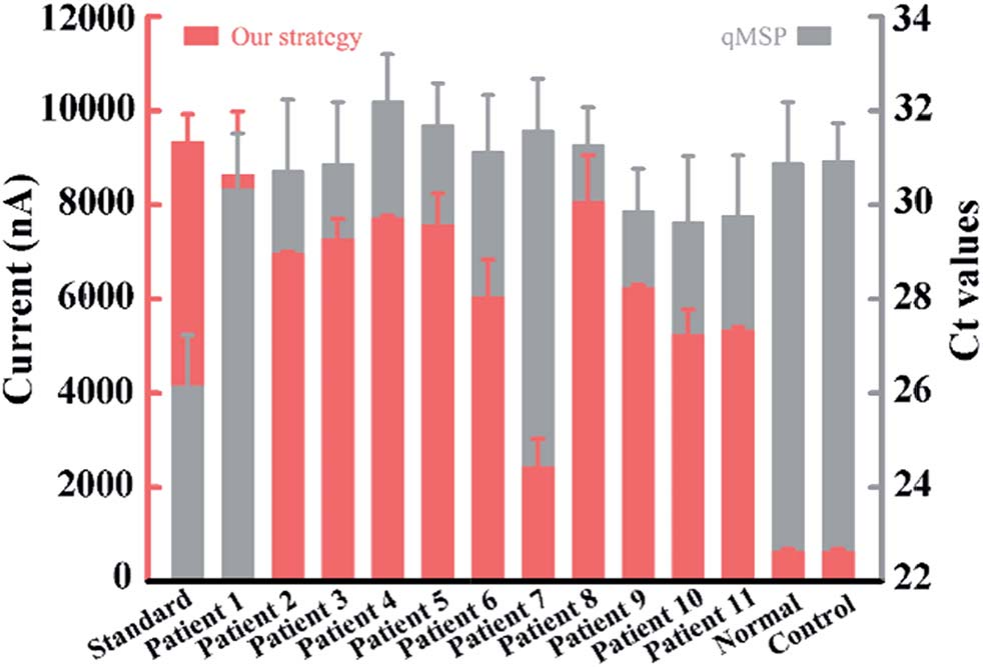
Clinical assay performance of circulating DNA extracted from 200 microlitres of plasma. The two colours represent data from our strategy and the qMSP.

To validate the application in trace amounts of clinical patient samples, circulating DNA extracted from 200 microlitres of NSCLC patients’ plasma was tested. Gratifyingly, all the eleven patient samples produced relatively much higher current values than those of the healthy volunteer and the negative control (Fig. 6), confirming the high methylation level of circulating DNA in these patients, whereas the conventional qMSP failed to detect the corresponding methylation pattern of these patients in such trace amounts of samples. These results show that our method has the ability to sensitively analyze trace clinical patient samples and the potential for DNA methylation detection-based early cancer diagnosis.

## Conclusions

In this work, a single molecule-sensitive electrochemical assay for tumor-specific circulating methylated DNA with ultrahigh specificity was reported based on a SEDA strategy. Single-copy methylated DNA in a clinical sample was accurately identified even in the presence of a 1000-fold excess of unmethylated alleles. The significant merits are ascribed to the integration of sequential discrimination–amplification processes, embodied in dual sequence discrimination events including methylation-specific annealing and specific interface hybridization, as well as cascade signal amplification processes represented as the AMSP and HRP catalytic reaction. The former eliminates the non-specific amplicons to reduce the background and the latter increases the signal. Notably, the AMSP dramatically reduced primer–dimer artifacts and the interfacial nanostructured probes significantly resist the non-specific adsorption of amplification byproducts. Additionally, the high S/B ratio enables the reduced use of PCR cycles, which allows for the further avoidance of non-specific amplification byproducts with positive feedback. Finally, while the conventional qMSP lacks this ability, the proposed strategy achieved the robust analysis of DNA methylation in trace amounts (200 microlitres) of plasma from lung cancer patients. Therefore, our single-copy sensitive electrochemical assay is superior, and we believe that it has an immediate and promising impact on fundamental research and clinical applications.
