# Supplementary material for: Single copy-sensitive electrochemical assay for circulating methylated DNA in clinical samples with ultrahigh specificity based on a sequential discrimination–amplification strategy
Source: Chem Sci. 2017 May 18;8(7):4764–70. doi: 10.1039/c7sc01035d (PMC5603958; doi:10.1039/c7sc01035d)
Supplement: Supplementary file 1 [file SC-008-C7SC01035D-s001.pdf]

Electronic Supplementary Material (ESI) for Chemical Science.
This journal is © The Royal Society of Chemistry 2017

Electronic Supplementary Information

Single copy-sensitive electrochemical assay for circulating methylated DNA in clinical samples with ultrahigh specificity based on a sequential discrimination-amplification system

Xuyao Wang,‡[a] Feng Chen,‡[a] Dexin Zhang,[b] Yue Zhao,[a] Jing Wei,[a] Lihua Wang,[c] Shiping Song,[c] Chunhai Fan[c] and Yongxi Zhao*[a]

[a] Key Laboratory of Biomedical Information Engineering of Education Ministry, School of Life Science and Technology, Xi'an Jiaotong University, Xianning West Road, Xi'an, Shaanxi 710049, P. R. China.
[b]Department of Respiratory Medicine, The Second Affiliated Hospital of Medical College, Xi'an Jiaotong University, Xiwu Road, Xi'an, Shaanxi 710049, P. R. China.
[c]Division of Physical Biology, Bioimaging Center, Shanghai Synchrotron Radiation Facility, CAS Key Laboratory of Interfacial Physics and Technology, Shanghai Institute of Applied Physics, Chinese Academy of Sciences, Shanghai 201800, P. R. China.

Email: yxzhao@xjtu.edu.cn

Experiment Section:

Materials and Reagents

All chemicals were obtained from commercial sources and used without further purification. Hot Start *Taq* DNA Polymerase and CpG methyltransferase (M.sssI MTase) were purchased from New England BioLabs (NEB). Tris(2-carboxyethyl) phosphine hydrochloride (TCEP) was purchased from Sigma-Aldrich (St. Louis, MO, USA). Horseradish peroxidase-conjugated avidin (avidin-HRP) was purchased from Roche Diagnostics (Mannheim, Germany) and diluted with 1% BSA (bovine serum albumin, Solarbio, Beijing, China) in 1× PBS. The TMB (3,3',5,5'-tetramethylbenzidine) substrate was purchased from Neogen in the format of a ready-to-use reagent (K-blue low activity substrate, H_2O_2 included). All solutions were prepared with Milli-Q water (resistivity = 18 MΩ cm) from a Millipore system.

DNA oligonucleotides

The thiol modified (C6 S-S) Tetra-B, C and D were supplied by Invitrogen. The specially designed Tetra-A and all other oligonucleotides were synthesized, and purified by Sangong. All the sequences are shown in Table 1, among which Tetra-A, B, C, D were used to form DNA nanostructured probes through self-assembling hybridization. The red region (34 bases) of the Tetra-A sequence was on the top of the tetrahedron in dissociative status and complementary to the green region of the target antisense ssDNA AT amplified by the biotin-labeled reverse primer (LR).

The buffer solutions involved in this study were as follows: The DNA tetrahedral nanostructure forming buffer was TM buffer (20 mM Tris base and 50 mM $MgCl_2$, PH 8.0). The washing buffer (W-buffer) for gold electrode was phosphate buffer saline (10 mM Na_2HPO_4, 2 mM KH_2PO_4, 37 mM NaCl and 2.7 mM KCl, PH 7.4). The hybridization buffer was H-buffer (10 mM Na_2HPO_4, 10 mM KH_2PO_4, 1 M NaCl, 20 mM $MgCl_2$) The TCEP solution was 30 mM in water. Buffer for electrochemical measurement was TMB substrate.

Instruments

All PCR were run on a LightCycler 480 (Roche).

An electrochemical workstation (CHI650E) was used for cyclic voltammetry and amperometry detection in a conventional three-electrode cell, including a platinum counter electrode, a reference electrode (Ag/AgCl, 3M KCl), and a gold working electrode. Cyclic voltammetry was performed from 0 to 0.7 V at a scan rate of 100 mV/s. Chronoamperometric detection was carried out at 100 mV, and the steady state of HRP redox reaction could usually be obtained within 100 sec.

Sample preparation

In vitro methylated DNA was obtained with M.SssI enzyme. Briefly, aliquots of 2 µl of 10× NEBuffer 2, 2 µl of S-adenosylmethionine (SAM, 3.2 mM), 4 U of M.SssI and 2 µg of DNA extracted from healthy volunteers were added to a final volume of 20 µl, followed by incubation at 37 ºC for an hour. The enzyme was then inactivated at 65 ºC for 20 min, and the solution was directly used for bisulfite treatment with an EZ DNA Methylation-Gold Kit (ZYMO Research) in accordance with the manufacturer's instructions. Final elution was performed with 20 µl of M-elution buffer. These bisulfite-treated DNA was aliquoted and stored at -20 ºC until ready for use.

Blood samples from NSCLC patients and healthy volunteers were obtained from The Second Affiliated Hospital of Xi'an Jiaotong University with written informed consent for DNA isolation and employment. All experiments were performed in compliance with the relevant laws and institutional guidelines.Blood samples were firstly centrifuged at 2000× g, and then supernatant plasma was carefully removed and collected. 200 µl of plasma was used for DNA extraction with a Serum/Plasma Circulating DNA Kit (Tiangen Biotech, Beijing, China). A final elution volume of 20 µl was used, followed by sodium bisulfite conversion as mentioned above. In order to yield more concentrated DNA, 10 µl of elution volume was recommended for circulating DNA from this real sample.

Self-Assembly of the DNA Tetrahedral Probes at Gold Electrode Surface.

The interfacial DNA nanostructured probes was derived from the previous published protocol.[1] We kept the sequence of the bottom three strands (Tetra-B, C and D, in Table 1) and lengthened Tetra-A sequence, yielding an appended pendant ssDNA probe to gain high hybridization selectivity. The lengthened 34 nt probe (Tetra-A highlighted in red) span the region (AT highlighted in green) which is directly extended by the labeled reverse primer with a 2 nt spare.

2.5 µl of four nucleotides (Tetra-A, B, C, D, each 20 µM initial concentration in TE buffer) were mixed in 5 µl of TM buffer with 5 µl of TCEP (30mM) to a final volume of 50 µl (30 µl water added), yielding the tetrahedral concentration of 1 µM. The solution was heated to 95 ºC for 5 min, then quickly cooled down to 4 ºC over 30 s and incubated until use.

The gold electrodes (Au, 2 mm in diameter) were cleaned according to the reported protocol. After drying with N_2 gas, 3 µl of the as-prepared tetrahedral solution were added to the surface and incubated overnight at room temperature. (Cap the electrodes with plastic electrode caps in order to prevent solutions from dying up.)

Primer design

This assay contraposed tumor-associated aberrant p16 methylation. The p16INK4a tumor suppressor gene that maps to chromosome band 9p21, is linked to the earliest stages of respiratory carcinogenesis.[2] This gene's hypermethylation status has been studied many times.[3-5] Thus, taken together these primers used, we designed the asymmetric MSP primers, 150F and LR (LR was rationally labeled with a biotin unit in its 5' terminal).

According to the known sequence of p16 gene promotor obtained from NCBI and the predicted bisulfite converted sequence through the bioinformatics program[6] (http://www.urogene.org/cgibin/methprimer/methprimer.cgi), we fixed the reverse primer at the original place (LR) and moved the forward primer closer step by step while considering the primer designing criteria in Oligo 7. Eventually we obtained three more forward primers (125F, 102F, 81F).

The template strand M was 58nt and the 3' dA terminal of M is a spacer. So, if not consider the dA overhang at the 3' end generated by the Hot Start Taq DNA Polymerase[7], the target amplicon is 57nt.

PCR condition

All PCR were run on a LightCycler 480 (Roche). To a 20 µl reaction volume, 7 µl of water, 2 µl of 10 × Standard *Taq* Reaction Buffer, 2 µl of dNTP (2mM), 2 µl of forward primers (200nM), 2 µl of reverse primers (2 µM), 1 µl of DNA and 2 µl of Hot Start *Taq* Polymerase were added and mixed well. Asymmetric PCR procedures (92 ºC for 15 sec, 64 ºC for 20 sec and 72 ºC for 15 sec) were performed for 50 cycles. After the PCR amplification, certain volume was taken out to mix with the H-buffer and the final volume was 5 µl for an electrode to hybridize with the probe.

For non-tail ssDNA target obtaining, 10 nM of strand M was added as the template with 200 nM of reverse primer (LR) in a 20 µl of PCR reaction system and thermo cycling on the LightCycler 480 with a normal APCR program.

Hybridization based AMSP product detection

AMSP product was mixed in a certain volume or by a certain dilution with the H-buffer. The electrode was incubated with 5 µl of the mixture for an optimized time at the room temperature and then rinsed by W-buffer. After dried with N_2 gas, the electrode was then incubated with 5 µl of avidin-HRP (1:1000 dilution in 1× PBS containing 1% BSA) for 15 min, followed by washing with W-buffer. After that, each electrode was detected by cyclic voltammetry (CV) and subsequent chronoamperometric measurement in the substrate of TMB.

Table S1 Sequences of the DNA oligonucleotides employed in this study

Name	Sequence (from 5' to 3')	Usage in this study
Tetra-A	ACATTCCTAAGTCTGAAACATTACAGCTTGCTACACGAGAAGAGCCGCCATAGTATTTTTTTTTTCGGGGAGTAGTATGGAGTTTTCGGTTGATTGGTT	The sequence to form the scaffold of the tetrahedron, containing a 34-nt appended probe
Tetra-B	SH-C_6-TATCACCAGGCAGTTGACAGTGTAGCAAGCTGTAATAGATGCGAGGGTCCAATAC	The sequence to form the scaffold of the tetrahedron
Tetra-C	SH-C_6-TCAACTGCCTGGTGATAAAACGACACTACGTGGGAATCTACTATGGCGGCTCTTC	The sequence to form the scaffold of the tetrahedron
Tetra-D	SH-C_6-TTCAGACTTAGGAATGTGCTTCCCACGTAGTGTCGTTTGTATTGGACCCTCGCAT	The sequence to form the scaffold of the tetrahedron
150F	TTATTAGAGGGTGGGGCGGATCGC	The forward primer for asymmetric PCR with an amplicon of 150nt
125F	TGCGTTCGGCGGTTGCGGAGA	The forward primer for asymmetric PCR with an amplicon of 125nt
102F	GGGAGAGTAGGTAGCGGGCGG	The forward primer for asymmetric PCR with an amplicon of 102nt
81F	CGGGGAGTAGTATGGAGTCGGC	The forward primer for asymmetric PCR with an amplicon of 81nt
LR	Biotin-GACCCCGAACCGCGACCGTAA	The reverse primer for asymmetric PCR
M	CGGGGAGTAGTATGGAGTTTTCGGTTGATTGGTTGGTTACGGTCGCGGTTCGGGGTCA	Template for the non-tail ssDNA
AT	Biotin-GACCCCGAACCGCGACCGTAACCAACCAATCAACCGAAAACTCCATACTACTCCCCGCCGCCGACTCCATACTACTCCCCGCCGCCCGCTACCTACTCTCCCCCTCTCCGCAACCGCCGAACGCACGCGATCCGCCCCACCCTCTAATAA	The 150 nt target amplicon

Note: The red region in Tetra-A is complementary to the green region in AT.

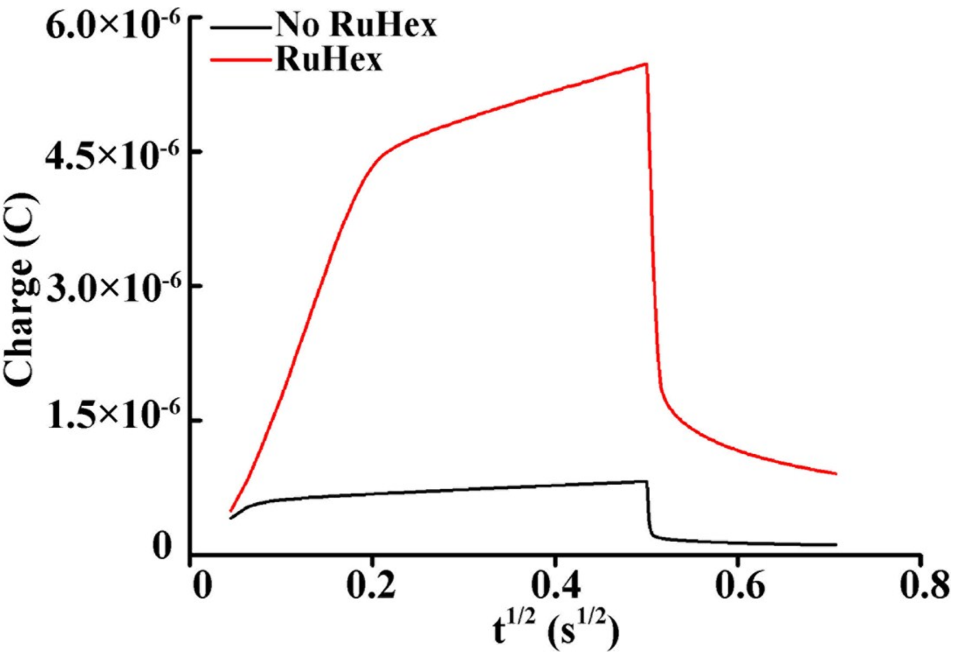

Fig. S1 Chronocoulometric curves of the immobilized DNA tetrahedron on the electrode. Briefly, the charge (Q) corresponding to RuHex electrostatically bound to surface-confined ssDNA is the difference of the intercept at t=0 obtained from plotting charge versus $t^{1/2}$ when RuHex exists and not exists. The surface density of DNA ϒ = $(QN_A/nFA)(z/m)$, where n is the number of electrons in the reaction, A is the area of the working electrode, m is the number of nucleotides in the DNA, z is the charge of the redox molecules and N_A is Avogadro's number.

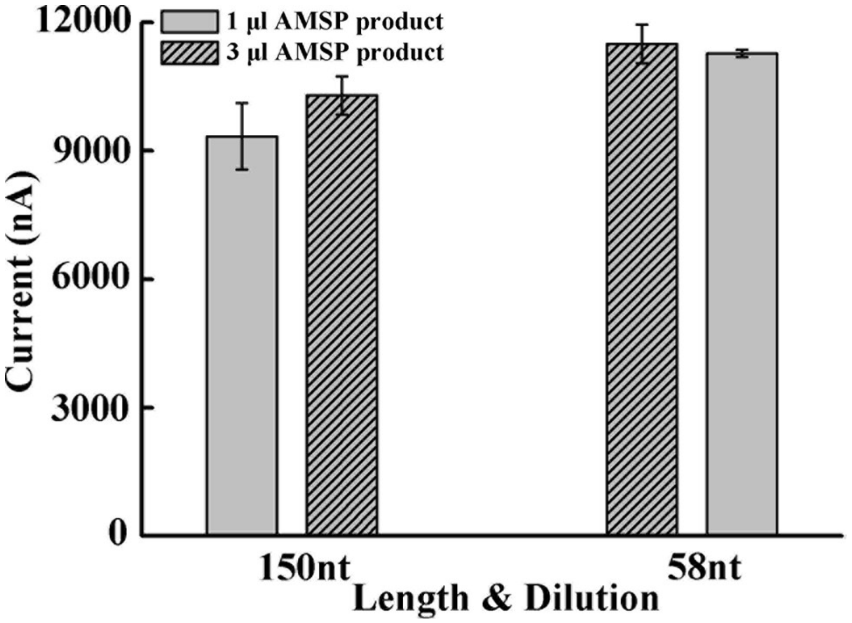

Fig. S2 Electrochemical responses on different concentrations and different lengths of the target ssDNA. 1 μl of the AMSP product is enough to analysis the result.

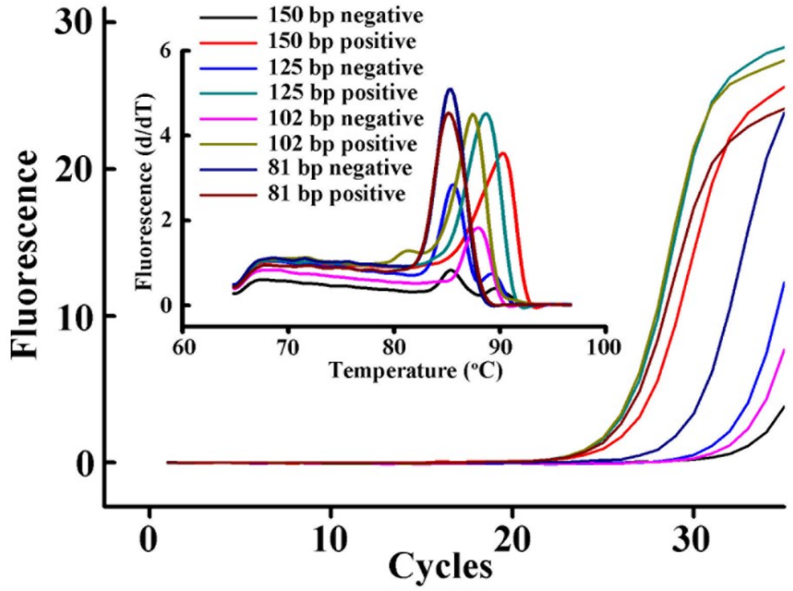

Fig. S3 qMSP curves and melting profiles of the four primer sets. “negative” represents the non-template control and “positive” has 15 ng of the methylated DNA in the reaction. The primer-dimer is unavoidable as the bisulfite conversion reduced the sequence complexity.

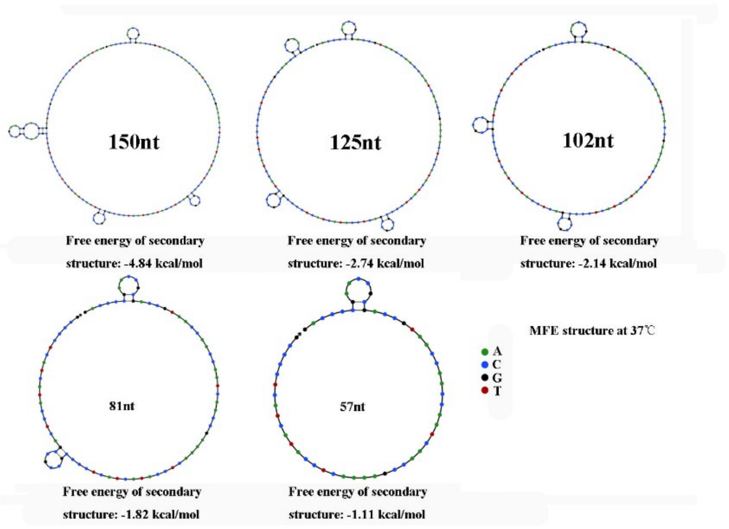

Fig. S4 Prediction of the secondary structure of the five target amplicon. The stem-loop secondary structure of these lengthy ssDNA is negligible and may not disturb the hybridization with the capture probe.

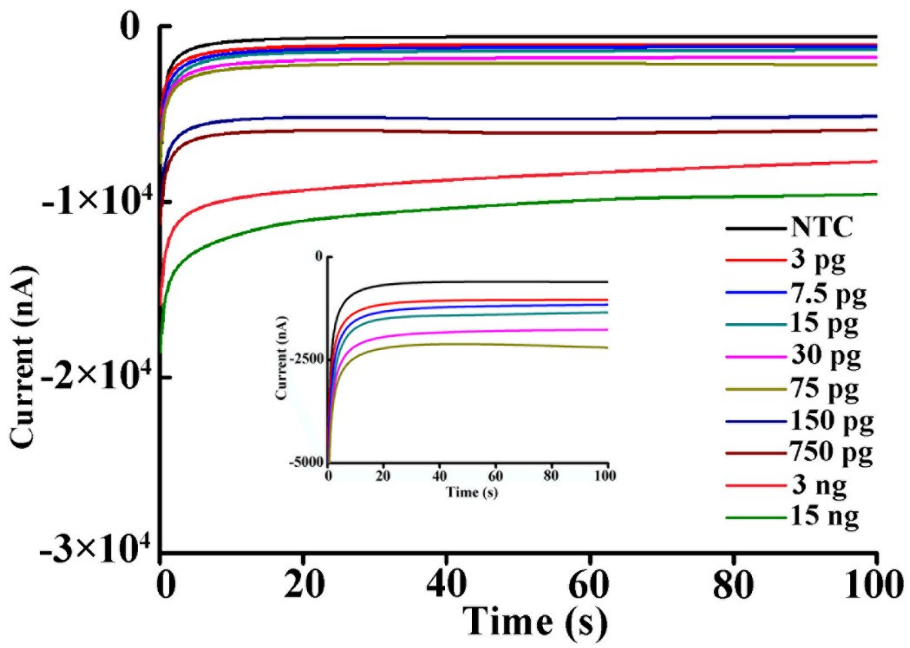

Fig. S5 Current values of different amount of the DNA input. The signal gradually rise as more DNA is added.

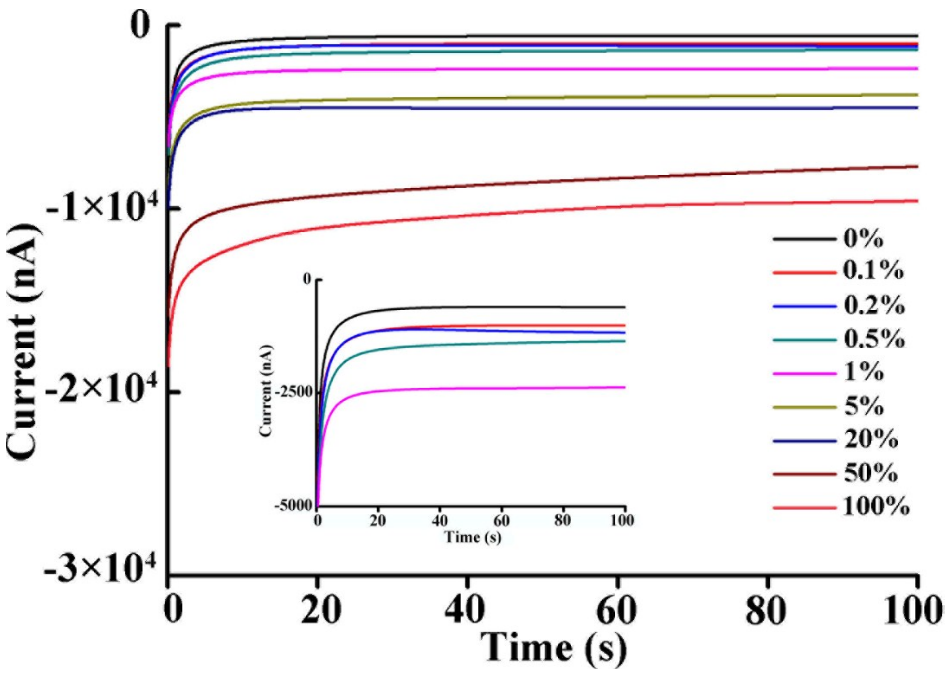

Fig. S6 Current values of different index of the methylated DNA input. The signal gradually rise as more methylated DNA is added.

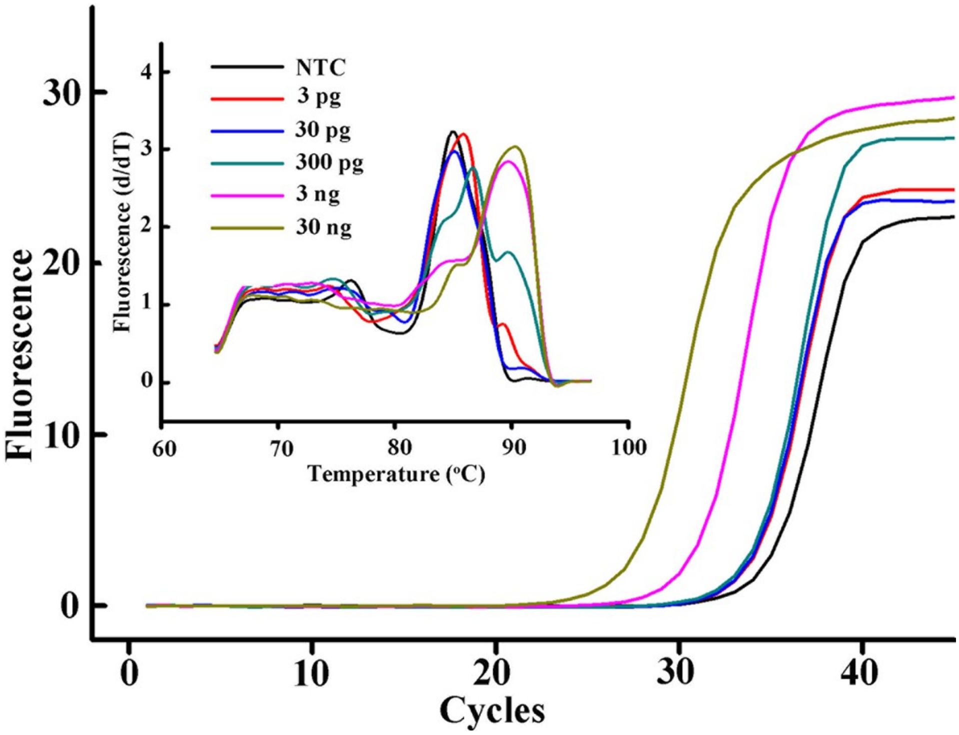

Fig. S7 qMSP curves and melting profiles of different amount of the DNA input. When more template DNA was added, the primer-dimer reduced and the target amplicon increased.

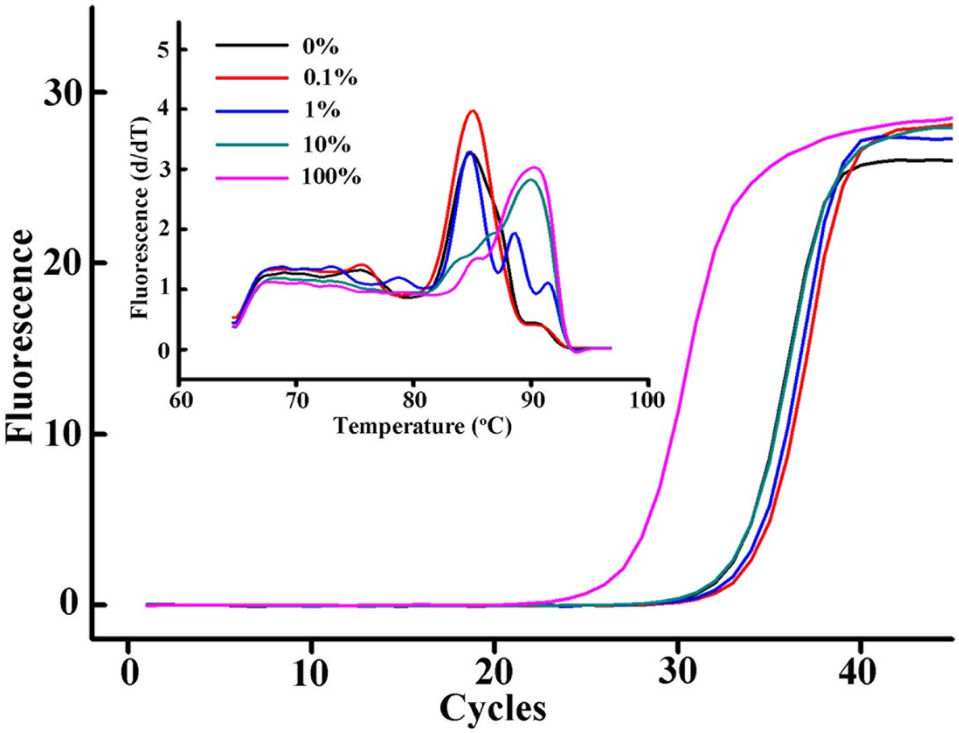

Fig. S8 qMSP curves and melting profiles of different index of the methylated DNA input. When more template methylated DNA was added, the primer-dimer reduced and the target amplicon increased.

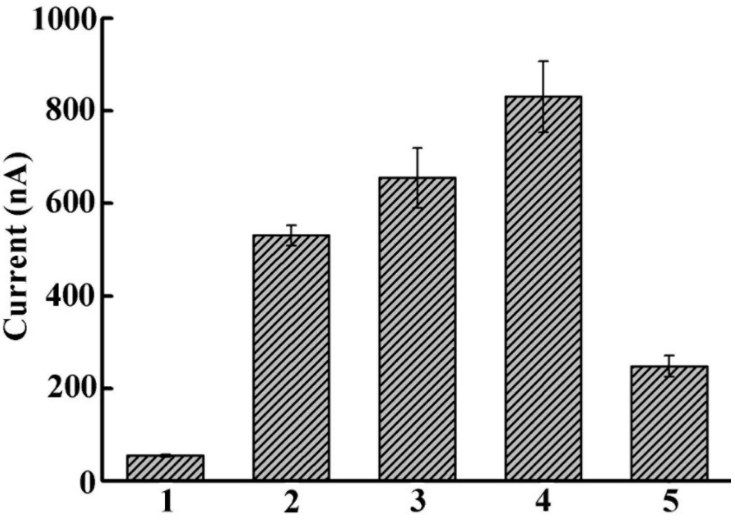

Fig. S9 Current values of some different background. (1) directly adding the avidin-HRP to the DNA tetrahedron (without the step of adding PCR product); (2) adding 1 µl of the APCR solution (not amplified); (3) adding 1 µl of the APCR solution (amplified); (4) adding 1 µl of the 2 µM reverse primer; (5) adding 1 µl of the symmetric PCR solution where the target is double-stranded DNA.

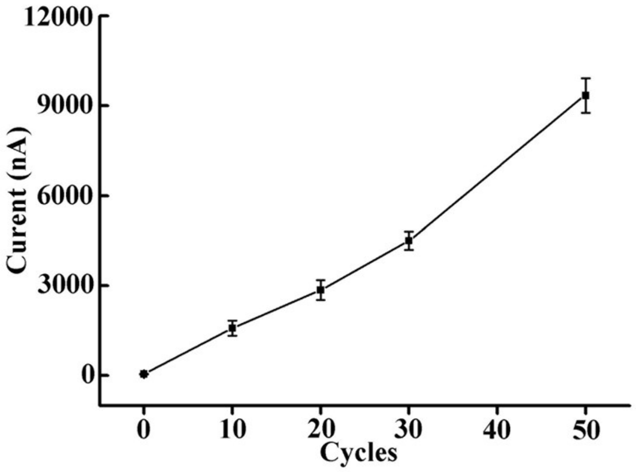

Fig. S10 Current values from different AMSP cycles. Reduced cyclers are able to be analyzed in our strategy.

References

1 M. Lin, P. Song, G. Zhou, X. Zuo, A. Aldalbahi, X. Lou, J. Shi and C. Fan, *Nat. Protoc.*, 2016, **11**, 1244-1263.
2 S. A. Belinsky, K. J. Nikula, W. A. Palmisano, R. Michels, G. Saccomanno, E. Gabrielson and J. G. Herman, *Proc. Natl. Acad. Sci. U. S. A.*, 1998, **95**, 11891-11896.
3 I. H. Wong, Y. D. Lo, J. Zhang, C. T. Liew, M. H. Ng, N. Wong and P. J. Johnson, *Cancer Res.*, 1999, **59**, 71-73.

4 C. Mund, V. Beier, P. Bewerunge, M. Dahns, F. Lyko and J. D. Hoheisel, *Nucleic Acids Res.*, 2005, **33**, e73.
5 Y. D. Lo, I. H. Wong, J. Zhang, M. S. Tein, M. H. Ng and N. M. Hjelm, *Cancer Res.*, 1999, **59**, 3899-3903.
6 L. C. Li and R. Dahiya, *Bioinformatics*, 2002, **18**, 1427-1431.
7 J. M. Clark, *Nucleic Acids Res.*, 1988, **16**, 9677-9686.
